# Effect of complex decongestive therapy on frailty and quality of life in women with breast cancer-related lymphedema: the before-and-after treatment study

**DOI:** 10.3389/fonc.2024.1297074

**Published:** 2024-05-24

**Authors:** Songül Keskin Kavak, Gamze Ünver

**Affiliations:** ^1^ Physical Therapy and Rehabilitation Clinic, Ankara Dr. Abdurrahman Yurtaslan Oncology Training and Research Hospital, Ankara, Türkiye; ^2^ Nursing Department, Faculty of Health Science, Kutahya Health Sciences University, Kutahya, Türkiye

**Keywords:** lymphedema, complex decongestive therapy, breast cancer, frailty, quality of life

## Abstract

**Objective:**

To investigate the impact of Complex Decongestive Therapy (CDT) on the severity of frailty and quality of life in individuals suffering from postmastectomy lymphedema syndrome.

**Methods:**

Participants who met the inclusion criteria were informed about CDT and informed consent was obtained. Edmonton Frailty Scale (EFS), extremity volüme (EV), lymphedema stage(LS), EQ-5D General Quality of Life Scale (EQ-5D-5L), and Quick Disabilities of Arm, Shoulder, and Hand (DASH) scores were evaluated by the same physician before and after treatment. A total of 15 sessions of CDT were performed for 3 weeks, five days a week. During the treatment period, hospitalized patients received guidance from a nurse on protecting the affected arm in their daily routine.

**Results:**

Eighty patients with breast cancer-related lymphedema who met the inclusion criteria were included in the study. Following a period of 3 weeks of practice and training, the specialist physician conducted the final evaluation and assessments. All patients showed a statistically significant reduction in EV, and regression in LS, EFS, and Quick DASH score (p<0.001). Statistically significant improvement was also observed in quality of life and general health status. (p<0.001).

**Conclusion:**

The application of 15 sessions of CDT and educational interventions to women with postmastectomy lymphedema syndrome due to breast cancer yielded positive outcomes. This approach led to an enhancement in patients’ functional capacity, improving their quality of life and a subsequent reduction in the severity of frailty.

## Introduction

Breast cancer is indeed a significant global health concern, ranking as the most common cancer among women worldwide. According to the 2020 GLOBOCAN data, it represents 11.7% of all cancer incidence in women, highlighting its widespread impact on female populations globally (1).

Breast cancer-related lymphedema (BCRL) is a chronic and progressive complication associated with treatments such as lymph node dissection, surgery, or radiation therapy for breast cancer. This condition involves the accumulation of protein-rich fluid in the interstitial spaces and results from the disruption of normal lymphatic drainage (2). Incidence rates for BCRL can vary widely, but estimates suggest that approximately 30% of women diagnosed with breast cancer may develop BCRL (3). Early detection and the implementation of appropriate management strategies are crucial in enhancing the quality of life for individuals affected by BCRL.

Frailty is characterized as a prominent geriatric syndrome arising from the progressive decline in physiological reserves within the neuromuscular, metabolic, and immune systems with advancing age (4). Predominant symptoms encompass weight loss, weakness, fatigue, and reduced mobility, whereas notable observations entail sarcopenia, osteopenia, malnutrition, compromised balance, and coordination, as well as deceleration in walking speed (5). Challenges in assessing frailty within the clinical routine of breast cancer patients are well recognized (6). Illustratively, in a study investigating the impact of pre-chemotherapy inflammation on post-chemotherapy frailty in breast cancer patients, it was observed that elevated levels of inflammatory cytokines before chemotherapy correlated with subsequent frailty following chemotherapy (7). Literature studies have provided substantiation to the notion that factors inducing immobilization, such as lymphedema, sarcopenia, and osteopenia, contribute to the heightened severity of frailty after breast cancer treatments (including surgery, chemotherapy, radiotherapy, and hormone therapy) (8, 9).

Research findings indicate that patients experiencing BCRL often encounter challenges such as restricted range of motion, joint stiffness, and a sense of apprehension towards movement, all of which contribute to a state of immobilization (10). Contemporary research has demonstrated that the asymmetrical increase in volume resulting from upper extremity lymphedema significantly impacts postural stability. This phenomenon can disrupt the swing phase of gait, leading to a compromised sense of balance and coordination. Consequently, these factors collectively contribute to a reduced quality of life for individuals experiencing such conditions (11).

The physical therapy intervention for individuals with lymphedema is rooted in the principles of Complete Decongestive Therapy (CDT), encompassing several core components. These components entail diligent skin care, the application of manual lymphatic drainage, the utilization of bandaging techniques, and the incorporation of specific exercises. This comprehensive approach aims to alleviate the symptoms and manage the progression of lymphedema, ultimately improving the overall well-being of affected individuals (12). Lymphedema, once established, lacks a complete curative solution. If left untreated, it can result in progressive extremity volume enlargement, subsequently fostering a chronic inflammatory state and culminating in fibrotic changes. Consequently, the imperative for efficacious lymphedema management remains pivotal to mitigate these undesirable outcomes and enhance the overall quality of life for affected individuals (13).

A recently published study presented a new perspective on the treatment of lymphedema after breast cancer surgery. In the protocol presented in the study; Patients with breast cancer-related lymphedema were treated with CDT for at least 3 months, and surgical treatment was selected for those who did not show significant improvement. After surgery, all patients received 6 months of postoperative CDT consisting of continuous multilayer bandage compression therapy and multiple weekly MLD sessions. Patients were then switched to single-layer compression garments while weekly MLD sessions were gradually reduced and discontinued (14).

In one study, frailty in breast cancer patients aged 50 years and older was assessed using a modified frailty score derived from self-reported assessments and was associated with increased frailty during treatment and up to 6 months after treatment (15).

In the realm of breast cancer treatment research, while there exist studies scrutinizing the ramifications of frailty throughout the therapeutic journey, a notable absence persists in terms of inquiries into the impact of comprehensive decompression therapy on frailty severity and quality of life in individuals afflicted by lymphedema stemming from breast cancer surgery (16).

The primary objective of our study is to ascertain the influence of CDT on the severity of frailty and quality of life in patients afflicted with BCRL. We also aim to provide valuable information on patient management and therapeutic interventions that may benefit health practitioners and medical staff responsible for the care and treatment of lymphedema.

## Methods

### Study cohorts

The study enrolled female participants who had experienced BCRL, manifesting as unilateral upper extremity swelling, edema, stiffness, and restricted movement. These individuals had previously undergone breast cancer treatment, including surgery, chemotherapy, and radiotherapy. They presented at the oncological rehabilitation outpatient clinic and demonstrated adequate cognitive function(assessed by a physician)to comprehend the conducted tests. Exclusion criteria encompassed subjects with visual impairments, vestibular balance challenges, a history of stroke, prior orthopedic or neurological disorders, deep vein thrombosis, or skin infections such as erysipelas or cellulitis.

### Power analysis

Power Analysis Power analysis is used in medical research to determine the smallest sample size required to detect a clinically significant effect at a given statistical significance level. We used to posthoc power analysis program. According to the posthoc power analysis performed using the G*Power 3.1.9.2 program. The G Power analysis used a pre-determined effect size (Cohen’s d) value of 0.5. The alpha level (Type I error) was set at 0.05, and the power level was set at 0.95. Given that a balanced sample size was used in the study, 80 patients were selected. the actual power was found to be 0.984 with a 5% margin of error.

### Data collection tools

#### The international society of lymphology scale

The International Society of Lymphology scale is utilized for staging lymphedema, and each patient is assessed based on the following stages (17):

- Stage 0: This signifies a subclinical state where there is no apparent swelling despite impairments in lymph transport.
- Stage 1: There is an early onset of the condition with a visible accumulation of protein-rich tissue fluid. The swelling may exhibit pitting, meaning that pressing with the thumb causes an indentation that persists for some time. The swelling subsides with elevation of the affected limb.
- Stage 2: Increase in swelling that does not subside with elevation. Initially, pitting is evident, but over time, the tissue undergoes further proliferation and hardening, making pitting more difficult to induce.
- Stage 3: Tissues become harder (more fibrotic), and pitting is absent. There is a potential for further enlargement of the lymphedema, sometimes reaching extreme proportions. Additionally, skin changes such as thickening, hyperpigmentation, increased (deepened) skin folds, fat deposits, and warty overgrowths are present.

#### Quick DASH (Disabilities of Arm, Shoulder and Hand)

The Quick-DASH is a concise adaptation of the Arm, Shoulder, and Hand Disability Questionnaire, aimed at evaluating limitations in activity and participation related to upper extremity conditions. Consisting of 11 questions, the scale assesses challenges individuals face during their daily activities. Each response is ranked on a Likert scale from 1 to 5, reflecting varying degrees of difficulty. The scale underwent a validity and reliability study in the Turkish context in 2011, demonstrating its robustness and appropriateness for assessing such impairments (18).

#### Edmonton frailty scale

The Edmonton Frailty Scale (EFS), initially developed by Rolfson et al. (19) at the University of Alberta, Canada, to assess frailty in elderly populations, underwent a Turkish validity and reliability study conducted by Aygör in 2013. The scale encompasses 9 dimensions associated with frailty, as recognized in the Comprehensive Geriatric Evaluation framework. These dimensions encompass cognitive status, general health status, functional independence, social support, medication use, nutrition, mood, continence, and functional status. The scale comprises a total of 11 items. Cognitive status is evaluated using the ‘clock test,’ while functional performance is assessed through the ‘Timed Get Up and Go test’. The scale includes 11 items covering the 9 frailty dimensions. The overall score, derived from summing up the scores of all 11 items, is employed for scale evaluation. Scores can range from 0 (lowest) to 17 (highest), with an increasing total score indicating heightened frailty severity. The internal consistency of the “Edmonton Frailty Scale” was assessed by Aygör, yielding a Cronbach’s alpha coefficient of 0.75. In this present study, the Cronbach’s alpha coefficient for the EFS was calculated to be 0.771 (19, 20)

#### EQ-5D-5L (EQ-5D general quality of life scale)

The EQ-5D-5L general quality of life scale, developed by the EuroQol group in collaboration with the Western European Quality of Life Research Society in 1987, comprises two main components. The first component is the health profile for the day, encompassing five sub-dimensions: mobility, self-care, usual activities, pain/discomfort, and anxiety/depression. Each sub-dimension offers respondents five response options: “no problem,” “slight problem,” “moderate problem,” “severe problem,” and “extreme problem.” The second component involves a visual analog scale, wherein individuals assign a value between 0 and 100 to depict their current health status on a thermometer-like scale. Quality of life scores ranging from 0 to 100 are obtained from the VAS section, with higher scores indicating a more favorable perception of health. The study utilized the Turkish version of the scale, which has been translated into 171 languages by the EuroQol group. Although the scale lacks a Cronbach’s alpha value calculated by EuroQol, international studies have consistently reported Cronbach’s alpha values exceeding 0.80 (21).

### Research practice

In our study, eligible patients received comprehensive information regarding the proposed CDT, and their informed consent was obtained. These patients underwent an initial assessment conducted by a specialized physician. A total of 15 CDT sessions were subsequently administered, spanning a 3-week duration with sessions conducted on 5 days per week. In our center, CDT was applied as a treatment modality including manual lymph drainage (MLD), multilayer bandaging, compression garment, diaphragmatic and aerobic exercise, and skin care.

MLD was applied to all patients every morning. The neck and shoulder collectors were drained. The contralateral axillary lymph nodes were stimulated. The lymph class was directed from the diseased side to the unaffected side via an anterior axillo-axillary anastomosis. On the diseased side, the inguinal lymph nodes were stimulated and the lymphatic fluid was diverted from the upper quadrant to the lymph nodes on the same side via the axillo-inguinal anastomosis. This redirected lymphatic fluid from the congested area to the area with adequate lymphatic flow.

Multilayer bandaging was applied daily after MLD. It was started distally with the shortest length bandages. It was progressed by stretching in a proximal direction. After treatment, each patient wore a stage 2 upper limb compression garment. Diaphragmatic and aerobic exercise combinations consist of resistance and breathing exercises. These exercises increase sympathetic activity and cause contraction of smooth muscle in the lymphatic vessels. They increase intra-abdominal pressure. This creates a pumping effect in the thoracic duct. These effects increase lymphatic absorption. It also increases the conversion of lymphatic fluid into free circulation.

Neutral PH skin creams were used for skin care. After bathing, the skin was thoroughly dried between the fingers and moisturized with odor-free natural products.

**Figure 1:**
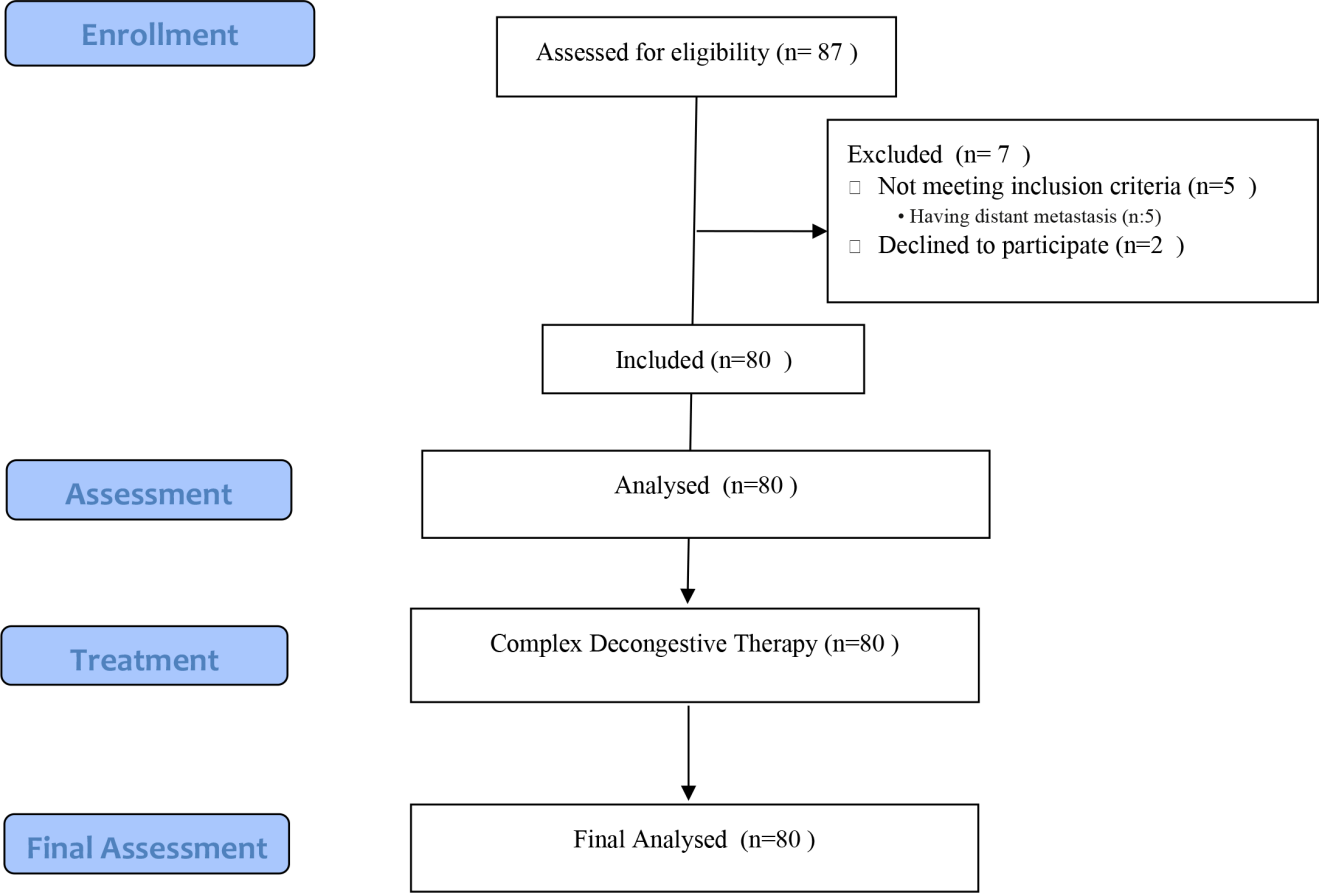
Flow chart of the study.

During the treatment period, patients who were hospitalized or under medical care were provided with guidance from a nurse on appropriate measures to safeguard their lymphedema-affected arms during their daily activities. Following three weeks of practice and guidance, a final evaluation was carried out, and the relevant assessment scales were completed by the attending physician (**Figure 1**).

### Analysis

The data analysis was conducted using IBM SPSS Statistics version 23 (IBM Corp., Released 2017). The normal distribution of the data was assessed using the Shapiro-Wilk test. Categorical data were presented as frequencies and percentages. For comparing dependent data that did not follow a normal distribution, the Wilcoxon Signed Ranks Test (Z*-table value) was employed. The Mann-Whitney U test (Z**-table value) was utilized to compare independent data sets that lacked a normal distribution. To compare measurement values across three or more independent groups without a normal distribution, the Kruskal-Wallis H test (X2-table value) was applied. A statistically significant threshold was defined as p < 0.05.

### Ethics committee approval

Individuals who fulfilled the eligibility criteria and sought care at the Health Sciences University Oncological Physical Medicine and Rehabilitation Department outpatient clinics were considered for participation in the study. Those who satisfied the inclusion and exclusion criteria expressed their willingness to partake, and provided written informed consent, adhering to the ethical principles outlined in the Helsinki Declaration, were enrolled in the study.

Ethical approval for this study was obtained from the Oncology Training and Research Hospital Clinical Research Ethics Committee (decision no: 2022-11/2113, date: 09.11.2022). Written informed consent was obtained from all patients after providing them with detailed information about the study.

## Results

The mean age of the participants was 65.42 ± 4.12 years. The majority of the participants were married (71.3%). Approximately 20% of the participants had a master’s degree or higher and a significant proportion were housewives. The mean body mass index(BMI) of the participants was 30.27 ± 4.45 kg/m^2^, indicating that they were significantly above the recommended weight range (classified as obese). Other sociodemographic characteristics of the patients are given in **Table 1**.

**Table 1:** Sociodemographic characteristics of the patients at baseline.

(N=80)		
Median age (range) — yr		65 (59 - 77)
Age group — no. (%)		
<65 yr		31 (38.75)
≥65 yr		49 (60)
Mean Height ± S.D.(range) — cm		159.75 ± 5.73 (144 - 169)
Mean Weight ± S.D.(range) — kg		77.01 ± 10.40 (56 - 103)
Mean BMI ± S.D.(range) — kg/m^2^		30.27 ± 4.45 (20.57 - 44.44)
Comorbidity— no. (%)		
Hypertension		18 (22.5)
Diabetes Mellitus		7 (8.75)
Hypothroidi		3 (3.75)
Asthma		8 (10)
Lymphedema Stages		
Stage 1		10 (12.5)
Stage 2		41 (51.25)
Stage 3		29 (36.25)
Duration of Lymphedema, Mean± S.D.(range) — month		
Marital status— no. (%)	Married	57 (71.3)
	Single	23 (28.8)
Educational status— no. (%)	Literate	11 (13.8)
	Primary education	46 (57.5)
	Secondary education	7 (8.8)
	Bachelor and above	16 (20.0)
Profession— no. (%)	Housewife	62 (77.5)
	Civil Servant/Retired	17 (21.3)
	Teacher	1 (1.3)
Smoking History— no. (%)	Current	34 (42.5)
	Never	46 (57.5)

BMI, Body Mass Index; S.D., Standard Deviation; cm, centimeters; kg, kilograms; yr, year.

The average duration of lymphedema in the patients was found to be 24.86 ± 27.10 months. Among the participants, 32.5% had hypertension and 26.3% had diabetes mellitus. All participants had undergone a consistent cancer treatment process. Notably, 100% of the patients reported experiencing symptoms of increased warmth and swelling in their extremities, with the majority (91.3%) also experiencing tension-related symptoms (**Table 2**).

**Table 2:** Findings regarding the health status of the patients.

Chronic disease — no. (%)	Hypertension	26 (32.5)
	DM	21 (26.3)
	Thyroid	8 (10)
	Asthma	5 (6.2)
	None	20 (25)
Cancer treatment process— no. (%)	Surgery	80 (100)
	Chemotherapy	80 (100)
	Radiotherapy	80 (100)
	Hormone therapy	80 (100)
Other symptoms in extremity— no. (%)	Tension	73 (91.3)
	Temperature rise	80 (100)
	Inflatable	80 (100)
	Pain	61 (76.3)
	Lethargy	45 (56.3)
	Feeling of heaviness	58 (72.5)
Mean Lymphedema time ± S.D.(range) — months		24.86 ± 27.10(1 - 96)

DM, Diabetes Mellitus; S.D., Standard Deviation.

The mean scores on the various scales were compared between the patients before and after the treatment. In comparison to the initial assessment, post-treatment evaluations revealed a reduction in extremity volüme (11), a decrease in lymphedema stage (15), an improvement in quality of life(Quick DASH), a decrease in frailty(EFS), a reduction in activity participation restrictions, and an enhancement in general health status(HSA) among the patients (**Table 3**).

**Table 3:** Comparison of the scales used in the research before and after.

	Before CDT	After CDT	p**	Z*
Extremity volume, Mean ± S.D. (range) — ml	680.5 (442.25-884)	287 (187-385.25)	**<0.001**	-7.772
Lymphedema stage Median. (range)	2 (2-3)	1 (1-1)	**<0.001**	-7.534
EQ-5D-5L,Mean± S.D. (range)	9 (7.25-10.75)	5 (5-6)	**<0.001**	-7.512
EFS, Mean± S.D. (range)	9 (4-13)	6.5 (3-9.75)	**<0.001**	-5.452
Quick DASH, Mean± S.D. (range)	56.81 (34.65- 72.72)	15.90 (6.81-22.72)	**<0.001**	-7.773
HSA, Mean± S.D. (range)	50 (40-57.5)	90 (80-90)	**<0.001**	7.850

Z*, Wilcoxon Signed Ranks Test; CDT, Complex Decongestive Therapy; ml, Milliliters; DASH, Disabilities of Arm, Shoulder and Hand; EFS, The Edmonton Frailty Scale; EQ-5D-5L, EQ-5D General Quality of Life Scale; HSA, Health status assessment. **p<0,05.

Among the different occupational groups, civil servants and retired patients experienced greater improvements in EFS, Quick DASH, and HSA compared to housewives. (p=0.001, p=0.001,p=0.034 respectively) In terms of marital status, married patients showed more pronounced improvements in HSA than single patients. (p=0.023) Moreover, patients without any chronic diseases exhibited more significant enhancements in EFS and EV. (p=0.014, p=0.001, respectively) (**Table 4**).

**Table 4:** Comparison of some sociodemographic characteristics of the patients and their post-treatment scale scores.

		EFS	LS	EQ-5D-5L	Quick DASH	HSA	EV
		Median (Q1-Q3)					
Profession	Housewife ^1^	7 (5-11)	1 (1-1)	5 (5-7)	19.3 (9.1-27.3)	80 (80 -90)	311 (206.3-394.3)
	Civil Servant/Retired^2^	4 (1-7)	1 (1-1)	5 (5-5)	6.8 (–15.9)	80 (80 -85)	187 (175.5-381.5)
	Teacher ^3^	–	1 (1-1)	5 (5-5)	**-**	90 (90 -90)	244 (244-244)
X^2^*		14.904	3.706	4.199	13.494	6.767	3.327
p**		**0.001**	0.151	0.123	**0.001**	**0.034**	0.189
Marital Status	Married	6 (3.5-9)	1 (1-1)	5 (5-6)	15.5 (6.8-27.27)	90 (80 -90)	287 (187-354.5)
	Single	8 (1-10)	1 (1-1)	5 (5-7)	15.9 (6.8-20.45)	80 (80 -90)	330 (187-432)
Z*		0.091	-0.635	-0.048	-1.312	-2.277	0.584
p		0.928	0.525	0.962	0.190	**0.023**	0.584
Chronical Disease	Yes	7 (4.25-11)	1 (1-1)	5 (5-6)	15.9 (9.7-27.27)	80 (80 -90)	312 (222-420)
	No	4.5 (1-8.75)	1 (1-1)	5 (5-6.5)	10.22 (–20.45)	80 (80 -90)	189 (166-246)
Z*		-2.453	-0.577	-1.174	-2.290	-1.648	-3.248
p**		**0.014**	0.056	0.24	0.022	0.099	**0.001**

X^2^*, Kruskal-Wallis H test; Z*, Wilcoxon Signed Ranks Test; CDT, Complex Decongestive Therapy; ml, Milliliters; DASH, Disabilities of Arm, Shoulder and Hand; EFS, The Edmonton Frailty Scale; EQ-5D-5L, The 5-level EQ-5D version; EV, Extremity volume; HSA, Health status assessment; LS, Lymphedema stage. **p<0,05.

## Discussion

Our study showed remarkable results in patients with BCRL undergoing CDT, demonstrating a reduction in limb volume, regression of LS, improvement in quality of life, reduction of frailty, alleviation of activity limitations, and an overall improvement in general health status.

Existing literature highlights that breast cancer patients often encounter physical and psychological challenges, exacerbated by the severity of lymphedema, leading to a negative impact on their overall quality of life (22, 23). For instance, Orhan et al. demonstrated in their study involving 83 breast cancer patients that quality of life and upper extremity functional status were more adversely affected in cases of severe lymphedema compared to milder instances. However, the overall population exhibited only a weak correlation between lymphedema severity and quality of life (24). In line with retrospective findings by Samancı et al., breast cancer patients receiving CDT over 15-30 sessions experienced a significant reduction in lymphedema volüme (25). In our study, statistically significant regression was also observed after 15 sessions of CDT.

In the study by Özcan et al., the group of patients who received CDT showed a reduction in lymphoedema volume, relief from pain and heaviness, and improvement in shoulder mobility. Functional capacity and quality of life were also improved in the study participants (26). Koul and colleagues carried out a lymphedema management approach that involved manual lymph drainage, complex decongestion therapy, and a home program with simple lymphatic drainage and exercise training. It is worth noting that the home program resulted in a 24% reduction in the cases studied by (27). In another study, patients with BCRL were divided into two groups conventional treatment and CDT, and significant improvement was observed in the health-related quality of life of patients after CDT (28). Our study was similarly consistent with the existing literature emphasizing that CDT contributes to improved quality of life.

Indeed, weight gain and obesity have been recognized as contributors to an elevated risk of lymphedema development (29). Despite observing elevated body mass indexes among the women in our study, it is noteworthy that the outcomes of complex decongestion therapy were not adversely impacted.

The emergence of frailty is influenced by factors such as sarcopenia, chronic illnesses, immune function alterations, and neuroendocrine system changes (30). In our study, where 75% of participants had chronic conditions and the mean age was 65, these elements likely contributed to the observed frailty severity.

Our study exhibited significant changes in the QUICK DASH scores and health status evaluations of the patients after the intervention. These results align with Devoogdt et al., who reported that 31% of breast cancer-treated patients experienced impaired shoulder mobility, and 51% faced limitations in their daily activities (31). The study found that women who self-reported symptoms of lymphedema had a significant higher score on the Disabilities of Arm, Shoulder, and Hand questionnaire (mean difference 23.4, 95% confidence interval 19.3–27.5). This higher score indicates that these women experienced more significant activity limitations and participation restrictions compared to those without lymphedema symptoms (32). Considering our study’s outcomes, the observed increase in QUICK DASH scores and improved functional capacities of patients suggest a potential enhancement in quality of life and a reduction in frailty severity.

## Limitations of the study

This study has several limitations. Firstly, there is no control group, the small sample size is a notable limitation, which can impact the generalizability of the findings to a larger population. Additionally, the lack of long-term follow-up limits the ability to assess the sustained effects of complex decongestive therapy. The method of assigning participants to treatment groups based on the day of the week they attended the clinic may introduce biases, and the absence of random assignment could be a potential limitation.

## Conclusion

In conclusion, the administration of 15 sessions of CDT along with educational interventions for breast cancer patients with BCRL resulted in improved functional capacity, subsequently enhancing their quality of life and thereby mitigating the severity of frailty. Future research should focus on evaluating the outcomes of a unified treatment plan in a larger and more diverse population to enhance the generalizability of the results.

## Data availability statement

The original contributions presented in the study are included in the article/supplementary material. Further inquiries can be directed to the corresponding author.

## Ethics statement

The studies involving humans were approved by ANKARA ONCOLOGY RESEARCH AND TRAINING HOSPITAL. The studies were conducted in accordance with the local legislation and institutional requirements. The participants provided their written informed consent to participate in this study.

## Author contributions

SK: Writing – review & editing, Project administration, Funding acquisition, Supervision, Investigation, Formal analysis, Data curation. GÜ: Resources, Writing – review & editing, Writing – original draft, Methodology, Formal analysis.

## References

[1] Sung H Ferlay J Siegel RL Laversanne M Soerjomataram I Jemal A. Global cancer statistics 2020: GLOBOCAN estimates of incidence and mortality worldwide for 36 cancers in 185 countries. CA Cancer J Clin. (2021) 71:209–49. doi: 10.3322/caac.21660 33538338

[2] Document C. The diagnosis and treatment of peripheral lymphedema: 2020 consensus document of the international society of lymphology. Lymphology. (2020) 53(1):3–19. doi: 10.2458/lymph.4649 32521126

[3] Muñoz-Alcaraz MN Jiménez-Vílchez AJ Pérula-De Torres LÁ Serrano-Merino J García-Bustillo Á Pardo-Hernández R. Effect of conservative rehabilitation interventions on health-related quality of life in women with upper limb lymphedema secondary to breast cancer: A systematic review. Healthcare. (2023) 11:2568. doi: 10.3390/healthcare11182568 37761765 PMC10531370

[4] Özdemir S Öztürk ZA Türkbeyler İH Şirin F Göl M. Geriatrik hastalarda farkli ölçekler kullanilarak kirilganlik prevalansinin belirlenmesi. Kahramanmaraş Sütçü İmam Üniversitesi Tıp Fakültesi Dergisi. (2017) 12(3):1–5. doi: 10.17517/ksutfd.338266

[5] Fairhall N Aggar C Kurrle SE Sherrington C Lord S Lockwood K. Frailty intervention trial (FIT). BMC Geriatrics. (2008) 8:27. doi: 10.1186/1471-2318-8-27 18851754 PMC2579913

[6] Jauhari Y Gannon MR Dodwell D Horgan K Tsang C Clements K. Addressing frailty in patients with breast cancer: A review of the literature. Eur J Surg Oncol. (2020) 46:24–32. doi: 10.1016/j.ejso.2019.08.011 31439357

[7] Gilmore N Kadambi S Lei L Loh KP Mohamed M Magnuson A. Associations of inflammation with frailty in patients with breast cancer aged 50 and over receiving chemotherapy. J Geriatr Oncol. (2020) 11:423–30. doi: 10.1016/j.jgo.2019.04.001 PMC6790284 30992181

[8] Suskin J Shapiro CL. Osteoporosis and musculoskeletal complications related to therapy of breast cancer. Gland Surgery. (2018) 7:411–23. doi: 10.21037/gs PMC6107589 30175057

[9] Vehmanen LK Elomaa I Blomqvist CP Saarto T. The effect of ovarian dysfunction on bone mineral density in breast cancer patients 10 years after adjuvant chemotherapy. Acta Oncologica. (2014) 53:75–9. doi: 10.3109/0284186X.2013.792992 23713891

[10] Can AG Can SS Ekşioğlu E Çakcı FA. Is kinesiophobia associated with lymphedema, upper extremity function, and psychological morbidity in breast cancer survivors? Turkish J Phys Med Rehabil. (2019) 65:139. doi: 10.5606/tftrd.2019.2585 PMC6706825 31453554

[11] Cuviena CF Perez CS Nardo VC Siqueira das Neves LM Rangon FB Guirro ECO. Influence of age and lymphedema on the postural balance of women undergoing breast cancer treatment. J Bodyw Mov Ther. (2021) 27:307–13. doi: 10.1016/j.jbmt.2021.02.024 34391250

[12] Tambour M Tange B Christensen R Gram B. Effect of physical therapy on breast cancer related lymphedema: protocol for a multicenter, randomized, single-blind, equivalence trial. BMC Cancer. (2014) 14:239. doi: 10.1186/1471-2407-14-239 24708851 PMC3978135

[13] Bennett JA Winters-Stone KM Dobek J Nail LM. Frailty in older breast cancer survivors: age, prevalence, and associated factors. Oncol Nurs Forum. (2013) 40:E126–E34. doi: 10.1188/13.ONF.E126-E134 PMC3988495 23615146

[14] Ciudad P Bolletta A Kaciulyte J Losco L Manrique OJ Cigna E. The breast cancer-related lymphedema multidisciplinary approach: Algorithm for conservative and multimodal surgical treatment. Microsurgery. (2023) 43:427–36. doi: 10.1002/micr.30990 36433802

[15] Magnuson A Lei L Gilmore N Kleckner AS Lin FV Ferguson R. Longitudinal relationship between frailty and cognition in patients 50 years and older with breast cancer. J Am Geriatrics Society. (2019) 67:928–36. doi: 10.1111/jgs.15934 PMC6490967 31034595

[16] Uslu A Canbolat O. Relationship between frailty and fatigue in older cancer patients. Semin Oncol Nurs. (2021) 37:151179. doi: 10.1016/j.soncn.2021.151179 34275706

[17] Witte MH Bernas MJ. Evolution of the 2020 international society of lymphology consensus document parallels advances in lymphology: an historical perspective. Lymphology. (2020) 53:1–2. doi: 10.2458/lymph.4650 32521125

[18] Koldas Dogan S Ay S Evcik D Baser O. Adaptation of Turkish version of the questionnaire Quick Disability of the Arm, Shoulder, and Hand (Quick DASH) in patients with carpal tunnel syndrome. Clin Rheumatol. (2011) 30:185–91. doi: 10.1007/s10067-010-1470-y 20411289

[19] Rolfson DB Majumdar SR Tsuyuki RT Tahir A Rockwood K. Validity and reliability of the edmonton frail scale. Age Ageing. (2006) 35(5):526–9. doi: 10.1093/ageing/afl041 PMC5955195 16757522

[20] Aygör H. Edmonton Kırılganlık Ölçeği'nin Türk toplumu için geçerlik ve güvenirliğinin incelenmesi: Ege Üniversitesi (2013).

[21] van Reenen M Janssen B. EQ-5D-5L user guide: basic information on how to use the EQ-5D-5L instrument. Rotterdam: EuroQol Res Foundation. (2015) 9.

[22] Abu-Helalah M Al-Hanaqta M Alshraideh H Abdulbaqi N Hijazeen J. Quality of life and psychological well-being of breast cancer survivors in Jordan. Asian Pacific J Cancer Prev. (2014) 15:5927–36. doi: 10.7314/APJCP.2014.15.14.5927 25081724

[23] Galiano-Castillo N Ariza-García A Cantarero-Villanueva I Fernández-Lao C Díaz-Rodríguez L Arroyo-Morales M. Depressed mood in breast cancer survivors: associations with physical activity, cancer-related fatigue, quality of life, and fitness level. Eur J Oncol Nurs. (2014) 18:206–10. doi: 10.1016/j.ejon.2013.10.008 24201014

[24] Orhan C Özgül S Nakip G Baran E Üzelpasaci E Çinar GN. Meme kanseri tedavisiyle ilişkili lenfödemi olan hastalarda lenfödem şiddetinin yaşam kalitesi, üst ekstremite fonksiyonu ve fiziksel aktivite düzeyi üzerindeki etkileri. Anadolu Kliniği Tıp Bilimleri Dergisi. (2019) 24:189–98. doi: 10.21673/anadoluklin.554019

[25] Samancı N Karataş Ö Samur A Çipli A Balcı N. Efficacy of complex decongestive therapy on breast cancer-related lymphedema: a cross-sectional study. J Surg Med. (2019) 3(4):300–3. doi: 10.28982/josam.551125

[26] Sezgin Ozcan D Dalyan M Unsal Delialioglu S Duzlu U Polat CS Koseoglu BF. Complex decongestive therapy enhances upper limb functions in patients with breast cancer-related lymphedema. Lymphat Res Biol. (2018) 16:446–52. doi: 10.1089/lrb.2017.0061 29356592

[27] Koul R Dufan T Russell C Guenther W Nugent Z Sun X. Efficacy of complete decongestive therapy and manual lymphatic drainage on treatment-related lymphedema in breast cancer. Int J Radiat Oncol Biol Phys. (2007) 67:841–6. doi: 10.1016/j.ijrobp.2006.09.024 17175115

[28] Melam GR Buragadda S Alhusaini AA Arora N. Effect of complete decongestive therapy and home program on health- related quality of life in post mastectomy lymphedema patients. BMC Women's Health. (2016) 16(23):1–9. doi: 10.1186/s12905-016-0303-9 PMC4855407 27145867

[29] Sudduth CL Greene AK. Current overview of obesity-induced lymphedema. Advances in wound care. (2022) 11(7):392–8. doi: 10.1089/wound.2020.1337 33493081

[30] Kapucu ss. Kırılgan yaşlı ve hemşirelik bakımı. Osmangazi J Med. (2017) 39:122–9. doi: 10.20515/otd.288967

[31] Devoogdt N Christiaens MR Geraerts I Truijen S Smeets A Leunen K. Effect of manual lymph drainage in addition to guidelines and exercise therapy on arm lymphoedema related to breast cancer: randomised controlled trial. BMJ. (2011) 343:d5326–d. doi: 10.1136/bmj.d5326 PMC3164214 21885537

[32] Dawes D Meterissian S Goldberg M Mayo N. Impact of lymphoedema on arm function and health-related quality of life in women following breast cancer surgery. J Rehabil Med. (2008) 40:651–8. doi: 10.2340/16501977-0232 19020699

